# Impact of neurovascular coupling and glymphatic function on cognitive function in asymptomatic cerebral small vessel disease

**DOI:** 10.3389/fneur.2026.1909936

**Published:** 2026-08-26

**Authors:** Jing Chen, Yifu Wang, Xing Chen, Caizhong Chen, Wenbin Shi, Jing Ding

**Affiliations:** 1 Department of Neurology, Zhongshan Hospital, Fudan University, Shanghai, China; 2 Department of Radiology, Zhongshan Hospital, Fudan University, Shanghai, China; 3 Health Examination Center, Zhongshan Hospital, Fudan University, Shanghai, China

**Keywords:** cerebral small vessel disease, cognitive function, diffusion tensor imaging analysis along the perivascular space index, glymphatic system, neurovascular coupling

## Abstract

**Introduction:**

Cognitive impairment ties to neurovascular coupling (NVC) dysfunction, which may interact with the glymphatic pathway. This study examined the relationships between NVC, glymphatic clearance, and cognitive function in asymptomatic cerebral small vessel disease (CSVD).

**Methods:**

In 231 asymptomatic CSVD patients, whole-brain NVC was assessed via voxel-wise ratios of cerebral blood flow to fractional amplitude of low-frequency fluctuations (CBF/fALFF) and spatial correlation; glymphatic function via the diffusion tensor imaging analysis along the perivascular space (DTI-ALPS) index. Partial correlation, linear regression (with covariates), and mediation analyses were used to explore associations with cognitive function.

**Results:**

CBF/fALFF ratio in precuneus and bilateral angular gyrus linked to DTI-ALPS index. Precuneus CBF/fALFF ratio correlated positively with episodic memory; CBF/fALFF correlation coefficients associated with executive function. DTI-ALPS index mediated the latter association (adjusted for demographics, vascular risks, and CSVD neuroimaging markers).

**Discussion:**

NVC may be a potential biomarker for cognitive dysfunction in asymptomatic CSVD. Optimizing NVC and glymphatic function could help prevent cognitive impairment in asymptomatic CSVD.

## 1 Introduction

Cerebral small vessel disease (CSVD) is an umbrella term for a group of disorders characterized by specific imaging findings on magnetic resonance imaging (MRI), with typical manifestations including lacunar infarction (LIs), white matter hyperintensities (WMH), cerebral microbleeds (CMBs), enlarged perivascular spaces (PVS), and brain atrophy (1). CSVD is particularly prevalent in older populations and is one of the main causes of increased risk for cognitive impairment and dementia (2).

Neurovascular coupling (NVC) refers to the dynamic interaction between the nervous and vascular systems, achieved through mechanisms such as vascular smooth muscle contraction/relaxation, blood flow regulation, and vascular permeability changes (3). NVC dysfunction severely impairs brain function, contributing to the development of cerebrovascular disease (4). Vascular events are closely linked to NVC dysfunction, compromising brain functional stability and cognitive ability (5).

The cerebral glymphatic system is responsible for the clearance of intracerebral metabolic waste products (6). It facilitates the transport of nutrients and sustains the homeostasis of the brain's internal environment (7). Dysfunction of the cerebral glymphatic system results in the accumulation of metabolic wastes, which induces neuronal damage and subsequent impairments in memory and executive function (8). It is closely linked to cognitive decline in vascular cognitive impairment and neurodegenerative conditions such as Alzheimer's disease (9). Diffusion tensor imaging analysis along the perivascular space (DTI-ALPS), a novel technique, is recognized as a metric for assessing the clearance function of the glymphatic system and CSVD pathophysiology by measuring diffusion capacity around deep medullary veins at the lateral ventricular body level (10).

In CSVD, glymphatic system and blood flow regulation are interconnected through their reliance on small vessel integrity and their roles in maintaining metabolic homeostasis (4). Small vessel dysfunction drives both the perivascular space (PVS)-mediated waste clearance deficits (detected by DTI-ALPS) and impaired blood flow regulation response to neural activity (detected by NVC) (11). Studies have reported that DTI-ALPS and NVC abnormalities predict cognitive impairment in CSVD (12, 13). A reduced DTI-ALPS index correlates with worse executive function and memory in CSVD patients, likely due to amyloid-βaccumulation in the hippocampus and prefrontal cortex (14). Blunted CBF responses to cognitive tasks (e.g., during working memory tests) predict poorer processing speed and attention, as neurons lack the resources to sustain activity (15). However, how DTI-ALPS and NVC interact with each other and influence cognitive function remains poorly understood in patients with asymptomatic CSVD, warranting further investigation.

In the current study, we proposed that lower NVC indicated the impaired blood flow regulation response to neural activity that would lead to lessen waste clearance in PVS, resulting in cognitive function decline in patients with asymptomatic CSVD. For cerebral blood flow to fractional amplitude of low-frequency fluctuations (CBF-fALFF) ratio, we first assessed the association between maps of CBF-fALFF ratio and the DTI-ALPS index. Subsequently, we analyzed the correlations between CBF-fALFF ratio in brain regions with significant associations and cognitive function, while also examining the role of the DTI-ALPS index within these correlations. For CBF-fALFF correlation coefficients, we initially explored its associations with cognitive function performance, and then investigated whether the DTI-ALPS index acts as a potential mediator in the relationship between NVC and cognitive function.

## 2 Methods

### 2.1 Participants

**Figure 1:**
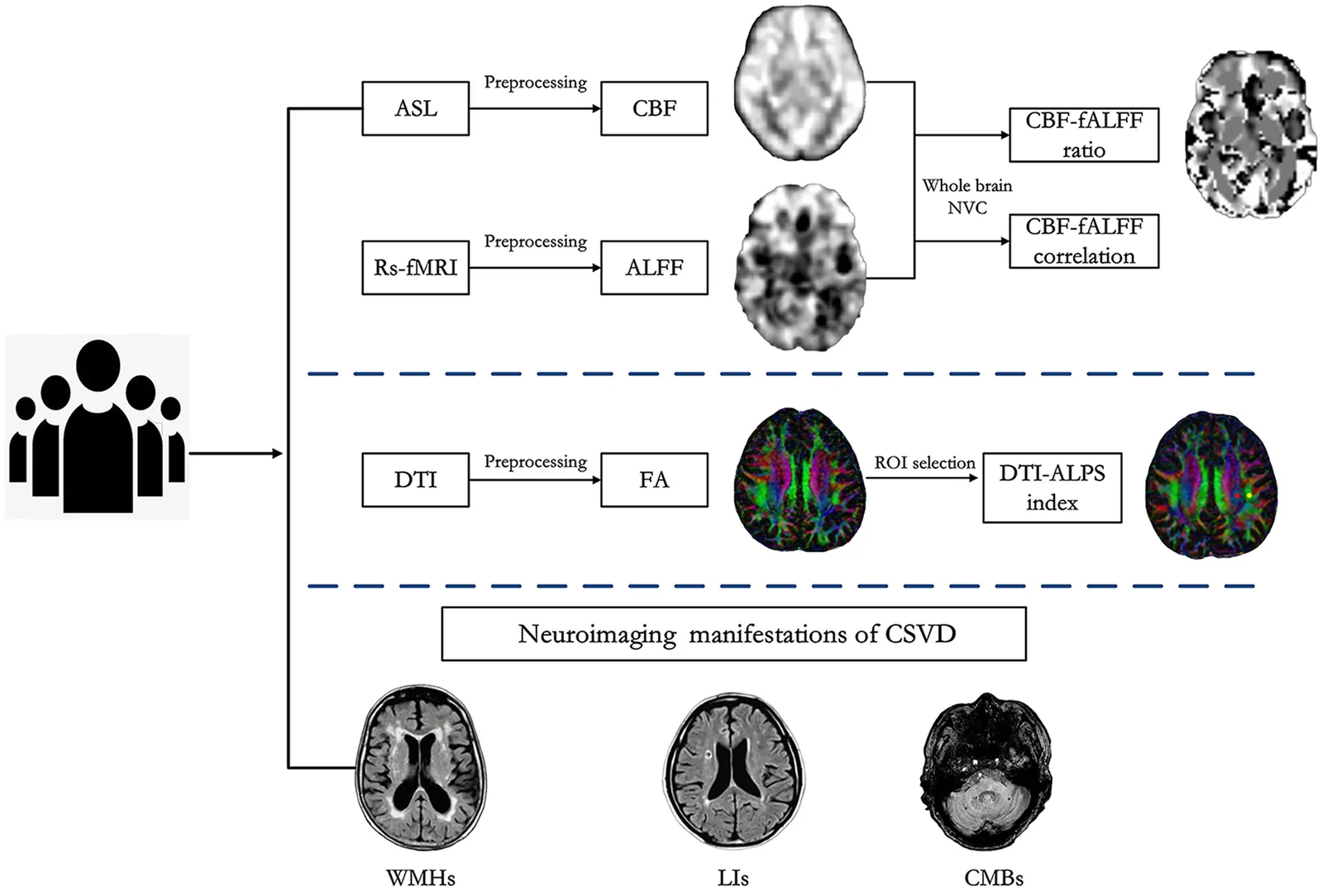
The flowchart of study design.

The procedures used in this study adhere to the tenets of the Declaration of Helsinki. Prior to participation, all eligible participants were provided with a detailed explanation of the study purpose, procedures, potential risks, and benefits. Written informed consent was obtained from each participant before any study-related procedures were performed. Between March 2018 and February 2020, consecutive adult subjects without overt cognitive impairment presenting for routine physical examinations at Zhongshan Hospital were prospectively evaluated for inclusion by a multidisciplinary panel comprising three senior neurologists and two radiologists. The specific inclusion criteria were as follows: aged between 50 and 85 years; no clinical signs or symptoms of neurological deficits detected by standardized neurophysical examination; presence of WMH of any severity in subcortical or periventricular regions on T2-weighted imaging (T2WI) or fluid-attenuated inversion recovery (FLAIR) sequences; ability to complete MRI scanning and neuropsychological assessments; and provision of written informed consent. The specific exclusion criteria included patients with concurrent brain pathologies (e.g., tumors, stroke), psychiatric disorders, or unstable medical conditions; those taking medications known to affect cognitive function (e.g., antipsychotics, antiepileptics); individuals with leukoencephalopathy of nonvascular origin (e.g., immune-mediated demyelination, metabolic/toxic etiologies, infectious processes); and those with contraindications to MRI (e.g., metallic implants) or severe claustrophobia. The study design flowchart is illustrated in Figure 1.

### 2.2 Cognitive function assessment

Cognitive function assessments were meticulously scheduled to be conducted in conjunction with participants' brain MRI scans, with all procedures completed within1week of enrollment. Global cognitive function was evaluated using the Mini Mental State Examination (MMSE) and the Montreal Cognitive Assessment (MoCA). All composite cognitive metrics were derived by converting raw test scores into Z scores. Executive function was gauged by averaging the Z scores from the inverted digit span test, Stroop C, and part B of the trail-making test. Information processing speed was assessed through the mean Z scores of part A of the trail-making test and Stroop B. Episodic memory was evaluated using the average Z scores from parts N4 and N5 of the auditory verbal learning test. Meanwhile, working memory and visuospatial function were estimated using the Z score from the sequential digit span test and the clock drawing test, respectively.

### 2.3 MRI data acquisition

MRI data were acquired using a 3.0-Tesla GE Discovery MR750 scanner (GE HealthCare, Waukesha, Wisconsin, USA) with a 32-channel head coil. Participants were secured with foam padding to minimize motion and provided earplugs to reduce noise. T1WI were obtained via a 3D MPRAGE sequence (TE = 3.1 ms, TR = 7.4 ms, FOV = 256 × 256 mm, FA = 11°, matrix = 256 × 256, voxel size = 1 mm^3^, 196 sagittal slices). Resting-state fMRI used a gradient-echo EPI sequence (TE = 30 ms, TR = 2000 ms, FOV = 220 × 220 mm, FA = 90°, matrix = 64 × 64, 3-mm slices with 1-mm gap, 43 axial slices, 200 volumes). 3D-pCASL data had parameters: TR/TE = 4,600/10.5 ms, FOV = 240 × 240 mm, 2-mm slices, FA = 109°, 36 slices, post-label delay = 2,000 ms. DTI employed a diffusion-weighted spin echo EPI sequence (TR = 8,500 ms, TE = 94.6 ms, FA = 90°, FOV = 256 × 256 mm, matrix = 128 × 128, 2-mm slices, 64 slices, 61 diffusion directions at b = 2,000 s/mm^2^).Susceptibility-weighted imaging data were acquired using the 3D gradient echo (GRE) SWI sequence (TR = 35 ms, TE = 20 ms, FOV = 220 × 220 mm, matrix = 256 × 256, FA = 15°, 1-mm slices with on gap). T2WI used a single-shot FSE sequence (TR = 5,500 ms, TE = 90 ms, FOV = 256 × 256 mm, matrix = 256 × 256, 4-mm slices with 1-mm gap). T2-FLAIR images were acquired via a single-shot FSE sequence (TR = 5,500 ms, TE = 97 ms, FOV = 256 × 256 mm, matrix = 256 × 256, 5-mm slices, 22 slices, 1-mm gap). Participants were instructed to remain awake, eyes closed, and avoid structured thought during scanning.

### 2.4 Functional data preprocessing and analysis

Data preprocessing was performed with Statistical Parametric Mapping 12 (SPM12) (available at http://www.fil.ion.ucl.ac.uk) operated on MATLAB R2014b (MathWorks, Natick, MA) and the Resting-State fMRI Data Processing Assistant (DPARSF) toolbox (16). For functional images, the initial 10 volumes were excluded, and the remaining ones underwent correction for time shifts induced by head movement (with displacement or rotation restricted to < 2 mm or 2°). Normalization was implemented by aligning fMRI images with their corresponding T1-weighted anatomical images; these anatomical images were segmented and transformed into Montreal Neurological Institute (MNI) space via the Diffeomorphic Anatomical Registration Through Exponentiated Lie Algebra (DARTEL) algorithm. The normalized functional images were then resampled to a voxel size of 3 × 3 × 3 mm^3^ and subjected to spatial smoothing using a Gaussian kernel [full width at half maximum (FWHM) = 8 mm]. Subsequently, detrending was applied to eliminate signal drift. Nuisance variables were then regressed out, including white matter (WM) signals, cerebrospinal fluid (CSF) signals, and Friston's 24 head motion parameters. Frame-wise displacement (FD) was calculated as well, and participants with a mean FD greater than 0.2 mm were excluded from subsequent analyses.

Following the above procedures, the time series of each voxel were converted to the frequency domain using Fast Fourier Transform. Within the low-frequency range (0.01–0.08 Hz), the square root of the mean power spectrum was computed as the amplitude of low-frequency fluctuations (ALFF). The fractional ALFF (fALFF), defined as the ratio of ALFF to the amplitude across the entire frequency band, was derived and then converted into z-maps using Fisher's r-to-z transformation.

### 2.5 Perfusion data pre-processing and analysis

Absolute cerebral blood flow (CBF) maps (unit: mL/100 g/min) generated from 3D-pCASL data were preprocessed using SPM12. Initially, linear co-registration was performed to align the CBF maps with their corresponding 3D T1-weighted images. The co-registered 3D T1-weighted images were segmented into three tissue types: GM, WM, and CSF, with the deformation field for each participant being created in this step. Next, individual CBF images were transformed into the standard MNI space by applying the previously generated deformation field and resliced to a voxel size of 2 × 2 × 2 mm^3^. Using DPABI, each voxel's CBF value was standardized through normalization against the mean CBF of the entire brain. Lastly, spatial smoothing was applied to the standardized CBF images using a Gaussian kernel with a FWHM of 8 mm.

### 2.6 Neurovascular coupling quantification analyses

To quantify the efficiency of metabolic support per unit of neuronal activity, the CBF (as indices of cerebral perfusion)-to-fALFF (as markers of neural activity) ratio was computed for each participant, with perfusion metrics normalized to intrinsic neuronal oscillatory activity within the GM mask. For the quantitative assessment of alterations in NVC, a whole-brain correlation analysis was conducted across the entire GM, examining the relationship between CBF maps and fALFF maps. This analytical approach investigating CBF-fALFF correlation coeffcientss facilitates an exploration of the intricate interplay between neural activity and vascular responsiveness.

### 2.7 DTI-ALPS processing

**Figure 2:**
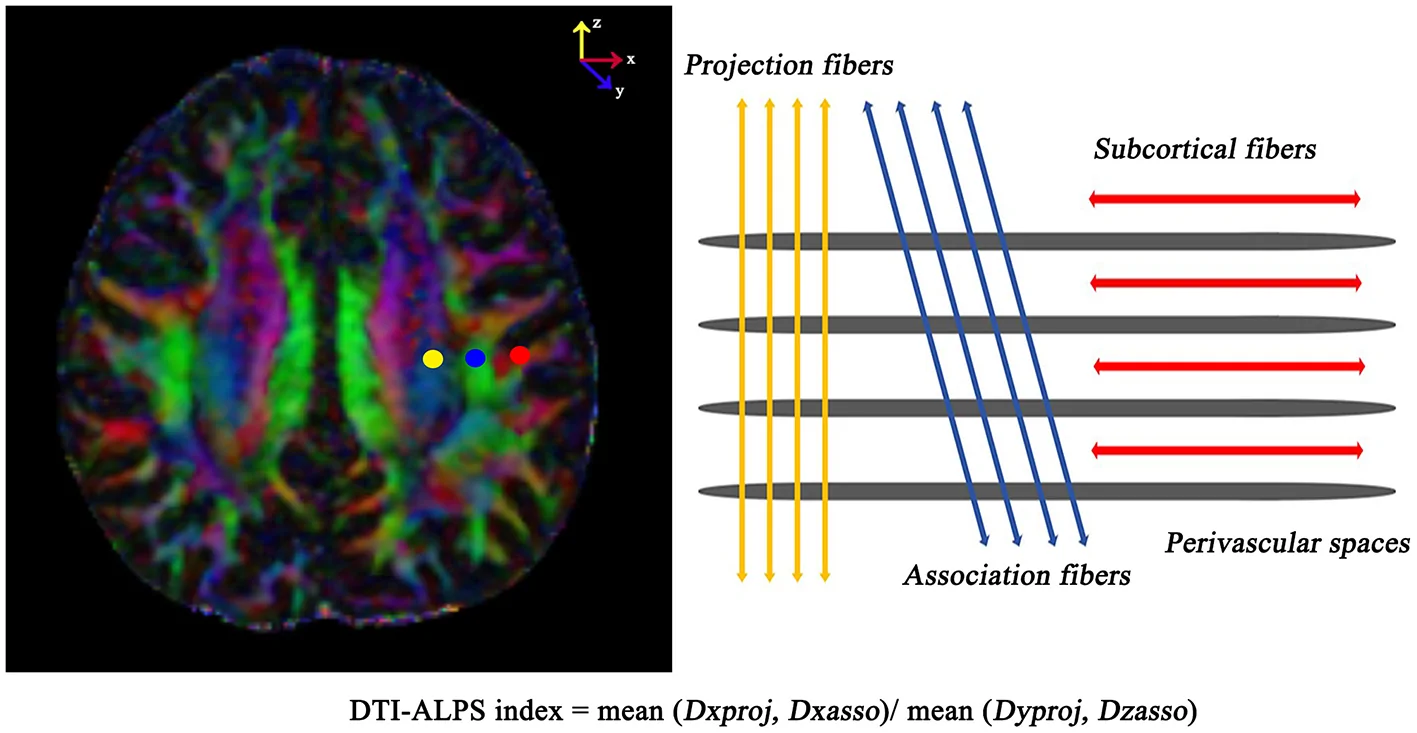
Analysis process for calculating the DTI-ALPS index. DTI-ALPS, diffusion tensor image analysis along the perivascular space.

The DTI-ALPS index was calculated as a previously published study (17). Briefly, DTI images were subjected to preprocessing steps, which included denoising, Gibbs unringing, correction of susceptibility-induced distortions, eddy current effects, and head motion artifacts. After tensor fitting, several diffusion metric maps were generated: color-coded fractional anisotropy (FA) maps, mean diffusivity (MD) maps, and diffusivity maps along the x-, y-, and z-axes (Dxx, Dyy, Dzz). These processed maps served as the basis for ALPS-Index calculation. Subsequently, four regions of interest (ROIs) with a 5-mm radius were manually delineated in the centrum semiovale on the color-coded FA map. The ROIs were positioned along projection and association fibers, oriented orthogonally to perivascular spaces at the level of medullary veins, with two ROIs on each side of the lateral ventricle. Diffusivity values along the x-, y-, and z-axes within these ROIs were measured separately for projection fibers and association fibers, labeled as X-proj, Y-proj, Z-proj (for projection fibers) and X-assoc, Y-assoc, Z-assoc (for association fibers). The ALPS-Index was computed as the ratio of mean diffusivity in specific directions:

ALPS-Index = $\frac{\text{Mean (Dxxproj, Dxxassoc)}}{\text{Mean (Dyyproj, Dzzassoc)}}$. Separate ALPS-Index values were calculated for the left and right hemispheres. For subsequent statistical analyses, the average of the bilateral ALPS-Index values was used. The Figure 2 demonstrated the analysis process for calculating the DTI-ALPS index.

### 2.8 Quantification of LIs, CMBs, WMH burden, and GM volume

LIs were assessed on T2-weighted and FLAIR images, defined as 3–15 mm cerebrospinal fluid (CSF)-filled cavities in white matter or subcortical regions (18). CMBs were identified on SWI as well-circumscribed focal low-signal areas < 10 mm in diameter. WMHs) were FLAIR-hyperintense lesions in cerebral white matter without central hypointensity (distinguishing them from LIs or PVS). WMH volume (WMHV) and total brain volume (TBV) were quantified via semi-automated NeuRoi software http://www.nottingham.ac.uk/research/groups/clinicalneurology/neuroi.aspx). WMHs were semi-automatically delineated by adjusting signal thresholds, interconnected across axial FLAIR images, and summed to compute total WMHV (19), which was then normalized to TBV (WMHV/TBV) and log-transformed for normality. Gray matter volume (GMV), including cortical gray matter, thalamus, and basal ganglia (caudate nucleus, putamen, pallidum, amygdala, accumbens), was measured via NeuRoi from smoothed images and normalized to TBV.

Two experienced neurologists independently quantified LIs, CMB counts, WMHV, and GMV. Discrepancies in LI/CMB counts were resolved by a third senior neurologist; WMHV/GMV analyses used the mean of the two initial assessments.

### 2.9 Statistical analysis

SPSS 24.0 was used for descriptive analysis of demographic characteristics, vascular risk factors (VRFs), cognitive-behavioral metrics, and imaging data. Data normality was assessed via the Kolmogorov-Smirnov test; continuous variables were expressed as mean ± standard deviation or median (interquartile range), while categorical variables were reported as counts and percentages. Skewed variables including WMHV and GMV were transformed using log10, and missing data points were imputed via the mean value method.

For analyses of CBF-fALFF ratio images, SPM12 was employed: a voxel-wise multiple regression analysis (within a gray matter mask) explored the relationship between CBF-fALFF ratio values and the DTI-ALPS index, with age, sex, and educational level included as covariates, and cluster-level family-wise error (FWE) correction applied (voxel-level *p* < 0.001; cluster-level *p* < 0.05 with a minimum cluster size of 50 voxels). Mean values from each significant cluster were extracted, and partial correlation analyses (adjusted for the same covariates) were performed to examine associations with cognitive function, with statistical significance defined as two-tailed *p* < 0.05 and Bonferroni correction applied to the correlation analyses.

Multiple linear regression analyses were used to examine the relationships between CBF-fALFF correlation coefficients and cognitive function indicators (executive function, information processing speed, episodic memory, working memory, and visual-spatial ability) with three sets of covariates controlled sequentially: Model 1 included demographic factors (age, gender, educational level); Model 2 built on Model 1 by incorporating VRFs (hypertension, diabetes mellitus, hyperlipidemia, BMI, smoking status, alcohol intake) to determine the incremental explanatory power of NVC for cognitive function beyond VRFs; Model 3 further added CSVD neuroimaging markers (LIs, CMBs, WMH burden, and GMV) to Model 2 to evaluate the contribution of these imaging markers. Additionally, mediation analyses were performed using the PROCESS macro in SPSS 24.0 (IBM Corporation, Armonk, New York, USA) to test whether the DTI-ALPS index mediated the association between NVC and cognitive function across all regression models, with the 95% confidence interval for the mediating effect calculated using 1,000 bootstrap samples; for bootstrap testing, the *p*-value of the indirect effect was determined as the minimum significance level α where the confidence interval derived from the bootstrap distribution excluded zero. False discovery rate (FDR) correction was applied across the five cognitive domains to control Type I error inflation owing to multiple comparisons.

## 3 Results

### 3.1 Demographics

A total of 1,025 potential participants were initially screened, with 231 individuals ultimately included in the final analysis. The study cohort had a mean age of 55.99 years, with males comprising 61.0% of the sample. The average educational duration was 9 years, ranging from 7 to 12 years. VRFs were distributed as follows: hypertension (42.0%), diabetes mellitus (14.7%), and dyslipidemia (53.7%). Additionally, 45.0% of participants reported a smoking history, and 47.2% had a history of alcohol consumption. With regard to CSVD neuroimaging markers, the median normalized WMHV was 4.18 × 10^−4^ (range: 2.05 × 10^−4^−8.59 × 10^−4^), the median normalized GMV was 3.99 × 10^−1^ (range: 2.50 × 10^−1^−4.05 × 10^−1^), the median number of LIs was 1 (range: 0 to 1), and the median number of CMBs was 2 (range: 1 to 2). The cohort had a mean DTI-ALPS index of 1.28. In terms of cognitive function, clinical assessments yielded a mean MMSE score of 28.19 and a mean MoCA score of 23.85. The median composite scores for cognitive domains were as follows: executive function (0.14), information processing speed (0.16), episodic memory (−0.04), working memory (0.17), and visual-spatial ability (0.29) (Table 1).

**Table 1:** Demographics, VRFs, CSVD neuroimaging markers, and cognitive status of the participants.

Demographics, VRFs, CSVD neuroimaging markers, and cognitive status of the Participants	Total (231)
Age, y	55.99 ± 7.82
Sex, male	141 (61.0)
Educational level, y	9.00 [7.00–12.00]
VRFs	
Smoking	104 (45.02)
Drinking	109 (47.2)
Hypertension	97 (42.0)
Diabetes mellitus	34 (14.7)
Dyslipidemia	124 (53.7)
BMI, kg/m^2^	24.58± 3.27
CSVD neuroimaging markers	
percentage of normalized WMHV, × 10^−4^	4.18 [2.05, 8.59]
percentage of normalized GMV, × 10^−1^	3.99 [2.50, 4.05]
LIs	1 [0, 1]
CMBs	2 [1, 2]
DTI ALPS index	1.28 ± 0.24
Cognitive function	
MMSE	28.19 ± 1.50
MoCA	23.85 ± 3.54
Executive function	0.14 [−0.39–0.53]
Information processing speed	0.16 [−0.44–0.42]
Episodic memory	−0.04 [−0.43–0.70]
Working memory	0.17 [−0.53–0.88]
Visual-spatial ability	0.29 [−0.41–0.29]

VRFs, vascular risk factors; CSVD, cerebral small vessel disease; BMI, body mass index; WMHV, white matter hyperintensity volume; GMV, gray matter volume; LIs, lacune infarcts, CMBs, cerebral microbleeds; MMSE, Mini-mental State Examination; MoCA, Montreal cognitive assessment; DTI-ALPS, diffusion tensor image analysis along the perivascular space.

Values are presented as mean (SD) or median [interquartile range] or *n* (%).

### 3.2 Multiple regression analysis and correlation analyses

**Figure 3:**
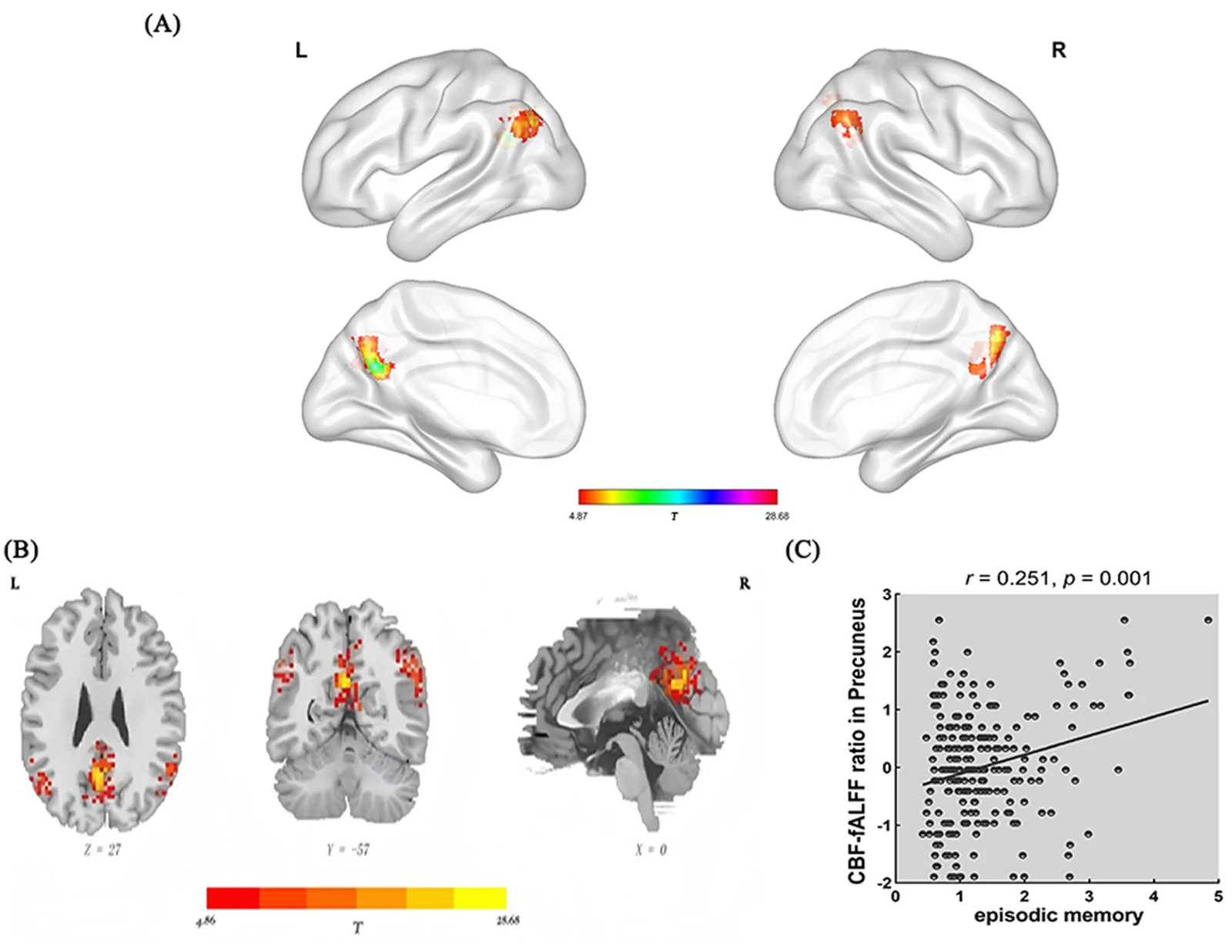
Brain regions showing significant correlations between the CBF-fALFF ratio and DTI-ALPS index, and correlations between CBF-fALFF ratio in precunues and episodic memory. **(A, B)** familywise error rate corrected (*p* < 0.05, cluster size ≥ 50 voxels). The color bar indicates the T value. **(C)** The correlations were uncorrected (*r* = 0.251, *p* = 0.001). CBF, cerebral blood flow; fALFF, amplitude of low-frequency fluctuation; DTI-ALPS, diffusion tensor image analysis along the perivascular space.

Multiple regression analysis revealed significant associations between CBF-fALFF ratio and the DTI-ALPS index in the precuneus, left angular gyrus, and right angular gyrus (Table 2 and Figures 3A, B). Further correlation analyses indicated that CBF-fALFF ratio in the precuneus was positively correlated with episodic memory (*r* = 0.251, *p* = 0.001, uncorrected) (Figure 3C). Nonetheless, subsequent to Bonferroni correction, this correlational relationship no longer attained statistical significance. In addition, no significant relationships were found between CBF-fALFF ratio in the left or right angular gyrus and cognitive functions, including executive function, information processing speed, episodic memory, working memory, and visual-spatial ability. Mediation analysis failed to identify a significant mediating role of the DTI-ALPS index in the association between the CBF-fALFF ratio in the precuneus and episodic memory.

**Table 2:** Brain regions with significant correlations between CBF-fALFF ratio and DTI—ALPS index.

Brain regions/BA	Peak MNI coordinates			Cluster voxels	Peak *t-*values
	X	Y	Z		
Precuneus/7	0	−57	27	414	27.18
Left angular/39	−45	−69	30	177	18.48
Right angular/39	57	−54	30	189	13.63

CBF, cerebral blood flow; fALFF, amplitude of low-frequency fluctuation; DTI-ALPS, diffusion tensor image along the long vascular space; BA, Brodmann's area; MNI, Montreal Neurological Institute.

### 3.3 Association between CBF-fALFF correlation coefficients and cognitive function

**Figure 4:**
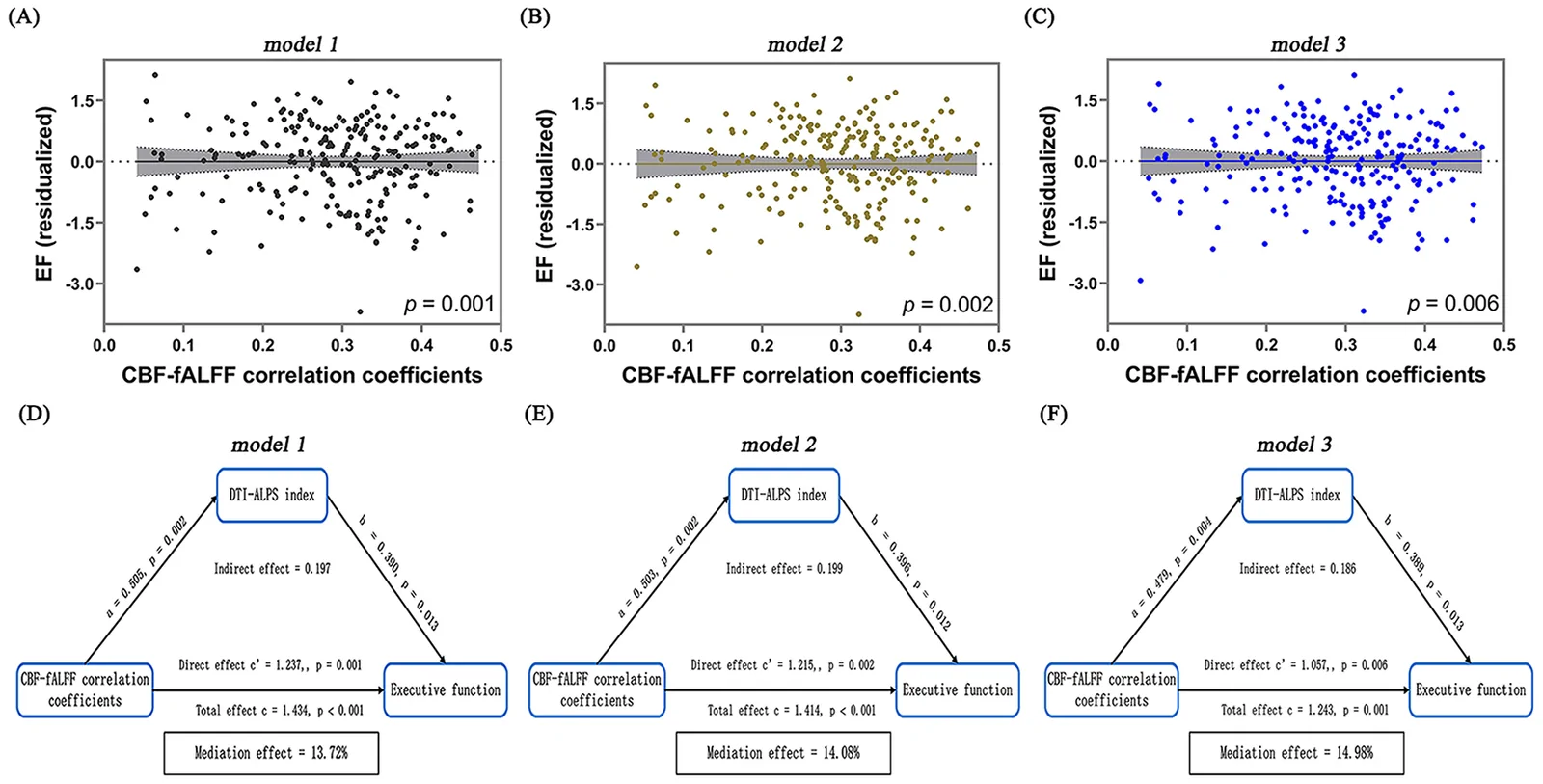
Associations between CBF-fALFF correlation coefficients and executive function, and mediation analysis investigating the relationship between these coefficients and executive function, with the DTI-ALPS index serving as the mediator. Scatter plots demonstrate a significant positive correlation between CBF-fALFF correlation coefficients and residualized executive function across multiple linear regression models: **(A)** Model 1, adjusted for demographic factors (age, gender, and education level; *p* = 0.001); **(B)** Model 2, which adds adjustment for vascular risk factors (diabetes, hypertension, hyperlipidemia, drinking status, and smoking status) to Model 1 (*p* = 0.002); **(C)** Model 3, which further incorporates neuroimaging markers of CSVD (WMHV, GMV, and numbers of LIs and CMBs) to Model 2 (*p* = 0.006). **(D)** Analysis with adjustment for demographic covariates (age, sex, and education level; Model 1); **(E)** Analysis incorporating additional vascular risk factors (diabetes, hypertension, hyperlipidemia, drinking status, and smoking status) into Model 1 as covariates (Model 2); **(F)** Analysis further including CSVD neuroimaging markers (WMHV, GMV, and counts of LIs and CMBs) into Model 2 as covariates (Model 3). CBF, cerebral blood flow; fALFF, amplitude of low-frequency fluctuation; CSVD, cerebral small vessel disease; WMHV, white matter hyperintensity volume; GMV, gray matter volume; LIs, lacunar infarcts and CMBs, cerebral microbleeds.

Demographic characteristics, VRFs, and CSVD neuroimaging markers were sequentially incorporated into the linear regression model as covariates. Linear regression analyses demonstrated that global NVC was independently linked to executive function scores across all three models: Model 1 (β = 1.43, *p* < 0.001), Model 2 (β = 1.41, *p* < 0.05), and Model 3 (β = 1.24, *p* = 0.001) (Tables 3, 4). The associations between CBF-fALFF correlation coefficients and executive function, after residualization for relevant covariates in each regression model, are visualized in Figures 4A–C. No significant associations between CBF-fALFF correlation coefficients and information processing speed, episodic memory, working memory or visuospatial ability were observed in linear regression analyses, and no significant indirect pathways via glymphatic function were identified in any adjustment model. Consistent conclusions were maintained after FDR correction for multiple comparisons. Detailed statistical results are provided in Tables 3, 4.

**Table 3:** Linear regression results for the association between CBF-fALFF correlation coefficients and cognitive function.

Cognitive function	Model	β	βstd	*R^2^*	Adjusted *R^2^*	Δ*R^2^*	95% CI	*P*
Executive function	1	1.434	0.220	0.255	0.242	0.255	0.696–2.172	< 0.001
	2	1.414	0.217	0.265	0.231	0.010	0.667–2.161	< 0.001
	3	1.243	0.192	0.297	0.251	0.032	0.495–1.998	0.001
Episodic memory	1	0.568	0.055	0.240	0.227	0.240	−0.622–1.759	0.348
	2	0.596	0.057	0.266	0.232	0.025	−0.570–1.788	0.326
	3	0.614	0.059	0.302	0.257	0.036	−0.582–1.810	0.312
Information processing speed	1	0.983	0.113	0.101	0.085	0.101	−0.101–2.067	0.075
	2	0.976	0.112	0.109	0.069	0.008	−0.124–2.075	0.082
	3	0.751	0.086	0.139	0.083	0.030	−0.361–1.863	0.184
Working memory	1	−0.805	−0.076	0.090	0.074	0.090	−2.133–0.523	0.233
	2	−0.845	−0.080	0.125	0.086	0.035	−2.171–0.482	0.211
	3	−1.095	−0.103	0.166	0.112	0.041	−2.427–0.238	0.107
Visual-spatial ability	1	0.773	0.073	0.012	−0.005	0.012	−0.612–2.157	0.273
	2	0.855	0.081	0.021	−0.024	0.009	−0.548–2.259	0.231
	3	0.742	0.070	0.028	−0.035	0.007	−0.698–2.181	0.311

Model 1: regressing out demographics.

Model 2: regressing out demographics and vascular risk factors.

Model 3: regressing out demographics, vascular risk factors, WMHV, GMV, Lis, and CMBs.

CBF, cerebral blood flow; fALFF, amplitude of low-frequency fluctuation; EF, executive function; DTI-ALPS, diffusion tensor image analysis along the perivascular space; CI, confidence interval; WMHV, white matter hyperintensities volume; GMV, gray matter volume; LIs, lacunar infarcts and CMBs, cerebral microbleeds.

**Table 4:** Mediation results for the association between CBF-fALFF correlation coefficients and cognitive function.

Cognitive function	Model	Path a			Path b			Direct effect			Indirect effect	
		β	95% CI	*P*	β	95% CI	*P*	β	95% CI	*P*	β	95% Boot CI
Executive function	1	0.505	0.192–0.817	0.002	0.390	0.084–0.696	0.013	1.237	0.491–1.983	0.001	0.197	0.030–0.510
	2	0.503	0.186–0.821	0.002	0.396	0.087–0.705	0.012	1.215	0.461–1.969	0.002	0.199	0.025–0.514
	3	0.479	0.154–0.803	0.004	0.387	0.084–0.694	0.001	1.057	0.302–1.815	0.006	0.186	0.022–0.504
Episodic memory	1	0.505	0.192–0.817	0.002	−0.102	−0.602–0.398	0.688	0.620	−0.599–1.839	0.318	−0.052	−0.399–0.167
	2	0.503	0.186–0.821	0.002	−0.126	−0.626–0.374	0.620	0.660	−0.561–1.880	0.288	−0.063	−0.401–0.163
	3	0.479	0.154–0.803	0.004	−0.126	−0.620–0.367	0.614	0.675	−0.546–1.896	0.277	−0.061	−0.386–0.143
Information processing speed	1	0.505	0.192–0.817	0.002	0.089	−0.366–0.545	0.387	0.938	−0.173–2.048	0.097	0.045	−0.237–0.482
	2	0.503	0.186–0.821	0.002	0.077	−0.384–0.538	0.743	0.937	−0.189–2.063	0.102	0.039	−0.255–0.502
	3	0.479	0.154–0.803	0.004	0.043	−0.415–0.502	0.852	0.731	−0.406–1.867	0.206	0.021	−0.263–0.428
Working memory	1	0.505	0.192–0.817	0.002	−0.001	−0.559–0.558	0.998	−0.804	−2.166–0.556	0.245	−0.001	−0.287–0.327
	2	0.503	0.186–0.821	0.002	−0.060	−0.617–0.496	0.830	−0.814	−2.173–0.544	0.239	−0.031	−0.369–0.256
	3	0.479	0.154–0.803	0.004	−0.097	−0.647–0.453	0.728	−1.048	−2.409–0.313	0.131	−0.047	−0.417–0.190
Visual-spatial ability	1	0.505	0.192–0.817	0.002	0.056	−0.526–0.638	0.850	0.744	−0.674–2.162	0.302	0.028	−0.242–0.293
	2	0.503	0.186–0.821	0.002	0.067	−0.522–0.656	0.824	0.822	−0.616–2.259	0.261	0.034	−0.237–0.294
	3	0.479	0.154–0.803	0.004	0.042	−0.552–0.636	0.812	0.722	−0.749–2.192	0.335	0.020	−0.260–0.268

Model 1: regressing out demographics.

Model 2: regressing out demographics and vascular risk factors.

Model 3: regressing out demographics, vascular risk factors, WMHV, GMV, LIs and CMBs.

CBF, cerebral blood flow; fALFF, amplitude of low-frequency fluctuation; DTI-ALPS, diffusion tensor image analysis along the perivascular space; CI, confidence interval; WMHV, white matter hyperintensities volume; GMV, gray matter volume; LIs, lacunar infarcts and CMBs, cerebral microbleeds.

### 3.4 Mediation effect of DTI-ALPS index on the relationship between NVC and the executive performance

Mediation analyses were performed with CBF-fALFF correlation coefficients set as the independent variable, executive function scores as the dependent variable, and the DTI-ALPS index acting as the mediator in all regression models. The findings demonstrated that the DTI-ALPS index played a significant mediating role in the association between CBF-fALFF correlation coefficients and executive function across the three models: Model 1 (indirect effect β = 0.197, *p* < 0.05, mediating ratio 13.72%), Model 2 (indirect effect β = 0.199, *p* < 0.05, mediating ratio 14.08%), and Model 3 (indirect effect β = 0.186, *p* < 0.05, mediating ratio 14.98%) (Table 3 and Figures 4D–F). Furthermore, a direct impact of CBF-fALFF correlation coefficients on executive function was detected in Model 1 (direct effect β = 1.237, *p* = 0.001), Model 2 (direct effect β = 1.215, *p* = 0.002), and Model 3 (direct effect β = 1.057, *p* = 0.006) (Table 3 and Figures 4D–F).

## 4 Discussion

Our study yielded findings that advance understanding of neurovascular and glymphatic interactions in asymptomatic CSVD. First, the CBF-fALFF ratio—a metric quantifying the amplitude of NVC responses to neural activity, correlated significantly with the DTI-ALPS index, a validated marker of glymphatic system clearance efficiency in two key brain regions (the precuneus and bilateral angular gyrus). Second, the CBF-fALFF ratio in the precuneus showed a positive association with episodic memory performance, hinting at region-specific links between NVC and cognitive function. Third, the DTI-ALPS index partially mediated the relationship between CBF-fALFF correlation coefficients (a measure of NVC strength, reflecting synchrony between CBF and neural activity) and executive function, suggesting a potential mechanistic pathway connecting vascular regulation, waste clearance, and cognitive decline.

To contextualize these findings, it is critical to clarify the biological significance of the metrics used. Derived from blood-oxygen-level-dependent (BOLD) fMRI signals, fALFF quantifies the relative contribution of low-frequency oscillations (0.01–0.08 Hz), a range closely tied to spontaneous neural activity to the total frequency spectrum (20). This makes fALFF a reliable index of neuronal oxygen uptake capacity, as low-frequency fluctuations align with the metabolic demands of resting-state neural networks. When paired with CBF measurements (from 3D-pCASL), CBF-fALFF analyses effectively characterize the coordination between vascular supply (CBF) and neural oxygen demand (fALFF), directly reflecting the functional integrity of the neurovascular unit. The complex of neurons, astrocytes, and microvessels that ensures brain activity is matched by adequate blood flow (21).

In the present study, we evaluated two complementary NVC metrics to capture different dimensions of neurovascular function: the CBF-fALFF ratio, which quantifies the “amplification” capacity of the vascular system in response to neural activity (a reduced ratio indicates blunted vascular responses, a hallmark of impaired NVC), and CBF-fALFF correlation coefficients, which measure the spatial synchrony between CBF and fALFF signals (higher values denote more robust coupling between neural activity and blood flow). This dual-metric approach is increasingly adopted in neuropsychiatric and cerebrovascular research, as it captures both the magnitude and consistency of neurovascular interactions (22). The DTI-ALPS index, by contrast, assesses glymphatic function by quantifying water diffusion along PVS surrounding deep medullary veins-spaces that serve as key conduits for glymphatic waste clearance. This method has been validated against the gold standard of intrathecal gadolinium-based glymphatic MRI, confirming that lower DTI-ALPS values correspond to reduced glymphatic clearance efficiency (23). Multiple prior studies have further linked reduced DTI-ALPS indices to CSVD-related radiological abnormalities (e.g., WMHs, LIs) and cognitive decline, underscoring its relevance as a functional marker of CSVD pathophysiology (24, 25).

The regional specificity of our findings-with significant correlations between CBF-fALFF ratio and DTI-ALPS observed in the precuneus and bilateral angular gyrus—aligns with the anatomical and functional roles of these regions in the default mode network (DMN), a core resting-state network critical for higher cognitive processes (26, 27). The precuneus, located in the medial parietal cortex, acts as an integrative hub within the DMN, orchestrating the convergence of self-referential processing, episodic memory retrieval, and mental imagery (28). Its strong functional connectivity to other DMN nodes, including the posterior cingulate cortex and medial prefrontal cortex, enables the coordination of internal mentation, such as reflecting on past experiences or constructing future scenarios (29). This integrative role makes the precuneus indispensable for episodic memory, as it integrates contextual details (time, place, emotion) to reconstruct past events (30). Accordingly, precuneus dysfunction, common in CSVD and Alzheimer's disease, often manifests as deficits in memory consolidation and spatial navigation (31). The angular gyrus, situated at the parieto-temporal-occipital junction, functions as a cross-modal processing node within the DMN, specializing in integrating verbal, visual, and spatial information (32). Its connectivity extends beyond the DMN to language and attention networks, allowing it to link external sensory inputs with internal knowledge frameworks, a key bridge between the DMN's internal focus and the external environment (33). This makes NVC disruptions in the angular gyrus particularly impactful for cognitive processes dependence on cross-modal integration, though our study did not detect significant correlations between angular gyrus CBF-fALFF ratio and cognitive function (likely due to statistical power or subtlety of effects). Collectively, these findings highlight the importance of targeting localized neurovascular dysfunction when investigating the neural substrates of cognitive decline in asymptomatic CSVD.

Our analysis of cognitive function, encompassing both global assessments (MMSE and MoCA) and domain-specific metrics (executive function, episodic memory, working memory), revealed nuanced relationships. While the precuneus CBF-fALFF ratio correlated with episodic memory (uncorrected), NVC (measured via CBF-fALFF correlation coefficients) showed a consistent positive association with executive function across three regression models (adjusting for demographics, VRFs, and CSVD imaging markers). Notably, the DTI-ALPS index mediated this NVC-executive function relationship, suggesting a potential mechanistic pathway. Impaired NVC reduces perivascular flow, compromising glymphatic clearance of metabolic wastes (such as amyloid-β and tau) and inflammatory factors; this accumulation then contributes to executive function deficits (34).

Differential associations between distinct NVC metrics and cognitive domains may arise from network architecture and domain-specific vulnerability. Regional NVC within default-mode network nodes (precuneus and angular gyrus) showed only an uncorrected nominal correlation between precuneal CBF-fALFF ratio and episodic memory, which vanished following Bonferroni correction. No significant cognitive correlates were detected for angular gyrus NVC. In contrast, global whole-brain NVC exerted a significant indirect effect on executive function mediated by glymphatic function indexed via DTI-ALPS. Executive function relies on extensive frontoparietal circuits and is particularly susceptible to diffuse microvascular dysfunction and impaired waste clearance in cerebral small vessel disease, which can be adequately captured by a global NVC measure. Episodic memory is anchored to discrete default-mode network regions, yet localized NVC fluctuations within isolated cortical nodes appear insufficient to sustain robust cognitive associations after strict correction. Such findings highlight that global rather than regional NVC may serve as a more sensitive marker for executive dysfunction mediated through glymphatic pathways.

The pathological cascade may caused by NVC dysfunction in CSVD involves interconnected processes: normally, increased neuronal activity triggers vasodilation and elevated CBF to meet metabolic demands, but in CSVD, NVC dysfunction breaks this coupling, leading to inadequate CBF responses despite sustained neuronal activation (35). This mismatch causes regional hypoperfusion in cognition-relevant brain regions (e.g., prefrontal cortex, hippocampus, default mode network), disrupting synaptic transmission and energy metabolism critical for memory, executive function, and information processing (36). Additionally, CSVD-associated vascular stressors damage microvascular endothelium, impairing vasodilatory signaling and promoting blood-brain barrier (BBB) leakage; leaked plasma components and pro-inflammatory molecules then trigger neuroinflammation, exacerbating neuronal injury and white matter disruption (37). The glymphatic system, reliant on perivascular flow for waste clearance, is also compromised by NVC dysfunction, leading to accumulation of toxic metabolites (e.g., amyloid-β, tau) that leading to neuronal damage and cognitive impairment (38). Also, the glymphatic system carries immune cells and inflammatory factors. Dysfunction of the glymphatic system might result in neuroinflammation and neuronal damage (39). These chronic hypoperfusion, BBB damage and neuroinflammation and oxidative stress may directly damage neurons and oligodendrocytes, and impair communication between brain regions, resulting in cognitive slowing and executive dysfunction. This pathway is supported by a growing body of preclinical and clinical evidence. In rodent models of chronic hypertension, early NVC dysfunction in the hippocampus and prefrontal cortex precedes the development of memory deficits and white matter lesions, and restoring NVC via endothelial-protective agents reverses these impairments (40). Similarly, mouse models with CSVD-like pathology show that NVC dysfunction reduces glymphatic clearance efficiency, with impaired perivascular flow correlating with tau accumulation and spatial memory deficits, effects that are ameliorated by enhancing glymphatic function (41). Clinically, a longitudinal study of 214 vascular cognitive impairment (VCI) patients found that reduced baseline CBF in the prefrontal and parietal lobes correlated with faster annual decline in executive function, independent of traditional VRFs (42). Another 2-year multimodal study of 120 mild cognitive impairment (MCI) patients (60 meeting VCI criteria) linked baseline NVC uncoupling (reduced BOLD-CBF correlation) to progressive executive function loss, with endothelial dysfunction (measured via exosomal Ang-1/VEGF ratio) amplifying this effect (43). These findings collectively position NVC as a key driver of CSVD-related VCI, acting through hypoperfusion, endothelial damage, and glymphatic impairment, with preclinical work establishing causality and clinical studies validating NVC's role as a prognostic marker and potential therapeutic target.

Finally, our findings align with the broader literature linking glymphatic dysfunction to cognitive decline. In CSVD, reduced DTI-ALPS indices consistently correlate with lower global cognitive scores and deficits in specific domains (executive function, memory) (44). This pattern mirrors findings in neurodegenerative diseases, including Alzheimer's disease, Parkinson's disease, and idiopathic normal pressure hydrocephalus, supporting the hypothesis that glymphatic failure is a common pathological pathway underlying cognitive impairment across diverse conditions (11–13, 45). The glymphatic system's role in clearing pathological proteins (amyloid-β, tau) and iron deposits is particularly critical. When the pathway disrupted, toxic accumulation triggers neurotoxicity and inflammation, while iron buildup impairs synaptic transmission (39). Compounded by reduced removal of inflammatory cells, this cascade drives progressive neuronal and white matter damage, ultimately leading to cognitive decline.

This study has several limitations. First, its cross-sectional design prevents definitive establishment of temporal or causal relationships among NVC, glymphatic function (assessed via DTI-ALPS index), and cognitive performance. Second, potential selection and generalizability limitations exist. Only 231 of 1,025 screened individuals were enrolled in this single-center cohort with a specific ethnic background and unique demographic characteristics (61.0% male, mean education of 9 years). Lower educational levels may reduce cognitive reserve and alter neuropsychological test performance, thereby constraining result extrapolation. Furthermore, exclusive enrollment of participants with white matter hyperintensities limits the generalizability of our findings to other CSVD subtypes and more diverse populations. Third, statistical caveats apply: the precuneus CBF-fALFF ratio-episodic memory correlation did not survive Bonferroni correction (risk of Type I error), indicating the association may be less robust and requiring replication in larger cohorts; while mediation analyses were significant, they relied on cross-sectional data, and unmeasured confounders (e.g., genetics, detailed lifestyle beyond smoking/alcohol) could affect results. Fourth, enlarged perivascular spaces (EPVS) was not quantified in this study. Though inversely correlated with DTI-ALPS, EPVS indicates structural perivascular space dilatation rather than the glymphatic functional deficits measured by ALPS, and the two cannot substitute for one another. Patients with dominant EPVS may have divergent injury profiles. Unassessed EPVS may lead to residual phenotypic heterogeneity. WMH-only enrollment limits generalizability to asymptomatic CSVD characterized by prominent EPVS, isolated CMBs or silent lacunar infarcts without evident WMH. Finally, the sample size limits subgroup analysis power (e.g., stratification by CSVD severity or vascular risk burden). Larger longitudinal studies are needed to validate findings and explore the longitudinal evolution of NVC, glymphatic function, and cognitive decline in CSVD.

This study has several limitations. First, its cross-sectional design precludes definitive conclusions about temporal ordering and causal relationships among NVC, glymphatic function (indexed by the DTI-ALPS index), and cognitive performance. Second, selection bias and limited generalizability should be noted. Only 231 of 1025 screened individuals were enrolled in this single-center cohort of a specific ethnic population with distinct demographics (61.0% male, mean education of 9 years). Educational attainment modulates cognitive reserve and neuropsychological test performance, constraining result extrapolation. Third, statistical limitations exist. The correlation between precuneal CBF-fALFF ratio and episodic memory did not survive Bonferroni correction, implying potential type I error; this finding requires validation in larger cohorts. Despite significant mediation results, analyses relied on cross-sectional data, and unmeasured confounders (e.g., genetics, lifestyle factors beyond smoking and alcohol) may bias the associations. Fourth, enlarged perivascular spaces (EPVS) were not quantified. Although EPVS correlate inversely with DTI-ALPS, they reflect structural dilatation of perivascular spaces rather than glymphatic dysfunction, and the two metrics are not interchangeable. Patients with prominent EPVS may exhibit distinct vascular injury phenotypes, and unmeasured EPVS could introduce phenotypic heterogeneity. Recruitment restricted to participants with white matter hyperintensities (WMH) also limits generalizability to asymptomatic CSVD subtypes featuring severe EPVS, isolated cerebral microbleeds, or silent lacunar infarcts without overt WMH. Furthermore, the DTI-ALPS index acts merely as an indirect surrogate. Despite validation for assessing perivascular water diffusion, it cannot directly measure glymphatic flow or solute clearance, including amyloid-β clearance. Moreover, cerebrospinal fluid biomarkers and amyloid/tau PET data were unavailable, so the hypothesized mechanistic link between glymphatic impairment and toxic metabolite accumulation remains speculative. Finally, the sample size lacked sufficient power for subgroup analyses stratified by CSVD severity or vascular risk burden. Larger longitudinal cohorts are needed to validate our results and delineate the trajectories of NVC, glymphatic function and cognitive decline in CSVD.

This study provides tentative evidence supporting NVC's potential as a biomarker for cognitive dysfunction in asymptomatic CSVD. By exploring NVC-cognition relationships in this population, it offers preliminary but meaningful data that NVC could serve as a clinically relevant indicator, aiding early detection of otherwise undetectable asymptomatic cognitive changes. Additionally, observed associations suggest optimizing NVC alongside enhancing glymphatic clearance may be a viable strategy to address cognitive impairment in these patients. While further research is needed to validate these hints and explore mechanisms, current findings lay a foundation for considering such targeted interventions to prevent or mitigate cognitive decline in asymptomatic CSVD, opening new directions for future clinical and translational studies.

## Data Availability

The raw data supporting the conclusions of this article will be made available by the authors, without undue reservation.
